# Supplementary material for: An automated framework for exploring and learning potential-energy surfaces
Source: Nat Commun. 2025 Aug 18;16:7666. doi: 10.1038/s41467-025-62510-6 (PMC12361387; doi:10.1038/s41467-025-62510-6)
Supplement: Supplementary file 1 — Supplementary Information [file 41467_2025_62510_MOESM1_ESM.pdf]

Supplementary Information for
‘An automated framework for exploring and learning potential-energy surfaces’

Yuanbin Liu[1], Joe D. Morrow[1], Christina Ertural[2], Natascia L. Fragapane[1], John L. A. Gardner[1], Aakash A. Naik[2,3], Yuxing Zhou[1], Janine George*[2,3], and Volker L. Deringer†[1]

[1]Inorganic Chemistry Laboratory, Department of Chemistry, University of Oxford, Oxford, UK
[2]Department Materials Chemistry, Federal Institute for Materials Research and Testing, Berlin, Germany
[3]Institute of Condensed Matter Theory and Solid-State Optics, Friedrich Schiller University Jena, Jena, Germany

*janine.george@bam.de
†volker.deringer@chem.ox.ac.uk

Supplementary Note 1: Computational details

Random structure searching (RSS)

The RSS method is employed to explore the potential energy surfaces of different materials. In each iteration, 10,000 initial random structures are generated using `buildcell`[S1,S2], which has been integrated into `autoplex`. Structural optimisation is performed using a preconditioned LBFGS algorithm[S3] to minimise the enthalpy until the residual forces fall below 0.01 eV Å^{-1}. We also incorporate Hookean repulsion in the optimisation process, ensuring that when atoms come too close (below a predefined threshold), a Hookean repulsive force acts to push them apart. This mechanism helps maintain physically reasonable structures. The diversity and importance of all training data are determined using the `CUR` algorithm and Boltzmann-weighted energy histogram sampling, respectively, following Ref. S4.

Key parameters of the initial random structures are as follows:

Ti–O. For the TiO_2 potential model, the stoichiometric ratio is fixed at 1:2, with the number of atoms per unit cell ranging from 6 to 24. In most TiO_2 crystals, the unit cell contains an even number of atoms; however, there are a few exceptions with an odd number of atoms, such as quartz-structured TiO_2. Therefore, in our random structure search, the initial random structures are generated with 80% even-numbered atomic configurations and 20% odd-numbered. For the Ti–O model, to encompass as many polymorphs as possible, we employ multiple sets of `buildcell` parameters during the search. The stoichiometric ratio varies within the range of Ti:O = 3:1 to 1:2. The exploration of the potential energy surface, covering different stoichiometries, is executed within a single unified workflow. An extended exploration over 50 iterations is illustrated in Supplementary Fig. 1.

SiO_2. For SiO_2, the initial random structures contain between 6 and 24 atoms, with a ratio of 4:1 for even-atom to odd-atom unit cells. Additionally, the initial structures are assigned a maximum number of symmetry operations of 4. For both the GAP@PBE and GAP@SCAN models, all parameters used in the RSS process are identical, except for the choice of the DFT methods used for labelling. The results for the energy convex hull in Supplementary Fig. 2 show how the potential approximates the lowest-energy crystalline structures.

Water. For the water molecular system, we adopt a fragment-based RSS strategy[S5] instead of the conventional atom-based approach. In this method, a single water molecule is treated as the smallest building block and is randomly placed within a simulation box to generate initial random structures. The initial random structures contain 4 to 10 water molecules. We apply up to 12 symmetry operations to the initial structures. Since the intramolecular geometry of water is already well-defined, the main objective of the RSS process is to discover the optimal intermolecular arrangement—most importantly, to learn the hydrogen-bonding network. As shown in Supplementary Fig. 3, the iterative updates of the potential enable the model to generate an increasing number of structures containing hydrogen-bonding networks.

Phase-change materials. When generating the initial random structures for GST, the per-atom volumes were informed by the reference densities of both the amorphous and crystalline phases. Based on this, we expanded the per-atom volume range by ±20%, allowing for a broader search space. Specifically, 40% of the structures are generated using the reference density of amorphous phases, while the remaining structures are based on the reference density of

crystalline phases. Due to the broad density range explored in our search, the densities obtained from these two approaches exhibit some overlap. The setup process for IST is similar to that of GST.

Assessment of potential data leakage

The RSS process begins by exploring the potential energy surface from randomly generated structures. When successful, it could partially recover structures that resemble experimentally known phases. To ensure the reliability of model accuracy evaluation, we examined potential data leakage by performing a symmetry-based comparison between training and test structures using `pymatgen`[S6] and `spglib`[S7]. Structural matching was carried out with the following parameters: a fractional length tolerance of 0.2, a site tolerance of 0.3 (expressed as a fraction of the average free length per atom), and an angular tolerance of 5°. The structures under comparison were first reduced to primitive cells and scaled to equivalent volumes. Supercell transformations were also attempted when appropriate.

Through this analysis, we identified the prototype structures of the three Si phases and several Ti–O phases that emerged during the search. For example, the rutile and anatase phases appeared in both training sets, whereas some phases such as TiO_2-B were not found in either dataset. However, we confirmed that there are no identical structures, at least in terms of atomic site positions, between the training and test datasets. Even when the same crystal structure type appears in both, the structures exhibit distinct distortions.

Molecular-dynamics (MD) simulations

The ab initio MD simulations for amorphous IST were carried out following the second generation Car–Parrinello method proposed by Kühne et al.[S8], employing the Quickstep module in CP2K[S9]. The electronic wavefunctions were expanded via Gaussian-type orbitals using a triple-ζ basis augmented with polarisation functions, and the electron density was represented by plane-wave expansions with an energy cutoff of 300 Ry. Scalar-relativistic Goedecker-type pseudopotentials[S10] and the PBE functional[S11] were chosen. The Brillouin zone integration was performed exclusively at the Γ point.

ML-driven MD simulations were performed using LAMMPS[S12] in the NVT ensemble for comparison against DFT or experimental data.

The structural models for amorphous GST and IST at 300 K were generated using an identical melt-quench protocol for both ab initio MD and ML-driven MD simulations. To be specific, the ab initio MD simulation for IST was performed using a simulation cell containing 216 atoms. In contrast, the ML-driven MD simulations used larger cells, comprising 1,002 atoms for IST and 1,001 atoms for GST. The initial structural models were first randomised at 3,000 K for 30 ps and then cooled to 1,000 K over 30 ps. The liquid models were equilibrated at 1,000 K for another 30 ps, and further quenched to 300 K over the next 30 ps. The structural models were held at 300 K for another 30 ps, from which trajectories during the last 20 ps were taken for structural analysis. A timestep of 2 fs was used throughout the simulations. For ring statistics, 200 structures were sampled from a single ab initio MD run, while ML-driven MD sampling involved three independent runs with 200 structures each (600 structures in total).

For modelling liquid water, we adopted a 192-atom simulation cell and carried out a long ML-driven MD simulation of 1 ns. The last 100 ps of the trajectory were used for data analysis. Previous literature has shown that 192 atoms are sufficient to accurately reproduce the structural characteristics of liquid water[S13,S14]. Radial distribution function and hydrogen-bond analyses were performed using `ovito` and the `MDAnalysis` Python packages[S15–S18], respectively. In addition, the `ovito` package was used under version 3.12.0, `MDAnalysis` under version 2.8.0, and Python under 3.10.13.

Training NequIP models

We used the `graph-pes` package[S19] to train a `NequIP` model on the resulting water RSS dataset[S20]. We randomly sampled 95% of the data as a training set and validated on the rest. We found that the accuracy of the model as a function of the number of parameters quickly saturates, and so used a relatively light model consisting of 3 message passing layers, each using internal representations described by `32x0e + 32x0o + 16x1e + 16x1o + 8x2e + 8x2o` and a linear self-interaction as proposed in SevenNet[S21]. Experimenting with various other hyperparameters led us to select a cutoff of 5.0 Å for the interatomic interactions, an initial learning rate of 0.01, and a `ReduceLROnPlateau` scheduler with a patience of 20 and a factor of 0.8.

Supplementary Figures

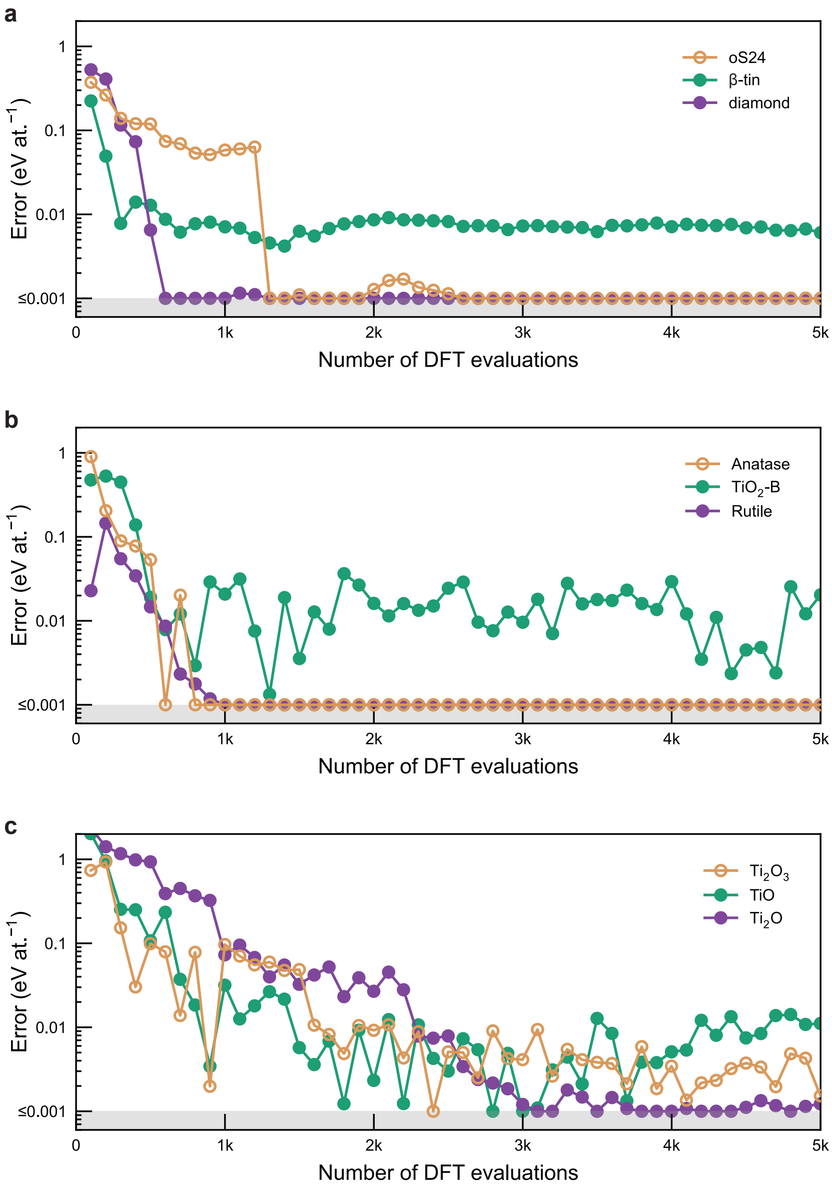

Supplementary Fig. 1: Automated GAP-RSS for representative materials. This figure supplements Fig. 2 in the main text, now showing GAP-RSS results up to 5,000 DFT evaluations in each case. As in the main text, we characterise selected modifications of: (**a**) elemental silicon; (**b**) TiO_2; and (**c**) binary Ti–O phases with different stoichiometric composition. Source data are provided as a Source Data file.

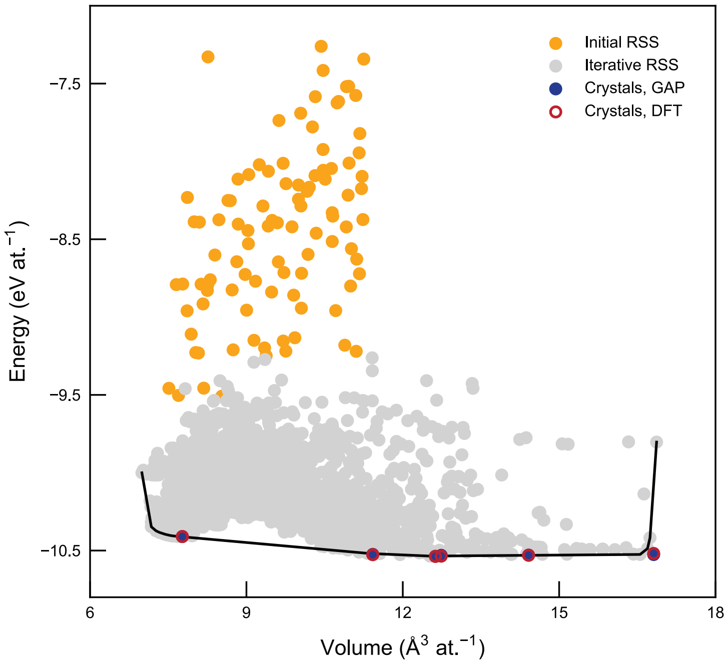

Supplementary Fig. 2: Energies as a function of volume per atom in the RSS dataset for SiO_2. This plot compares the energies of the initial randomised structures, RSS trajectories, and low-energy ice polymorphs. The black line represents the energy convex hull derived from all RSS structures. The iterative RSS process explores and samples structures starting from higher-energy regions and gradually moves toward lower-energy regions[S22], ultimately approaching the stability region of relevant crystalline polymorphs. Source data are provided as a Source Data file.

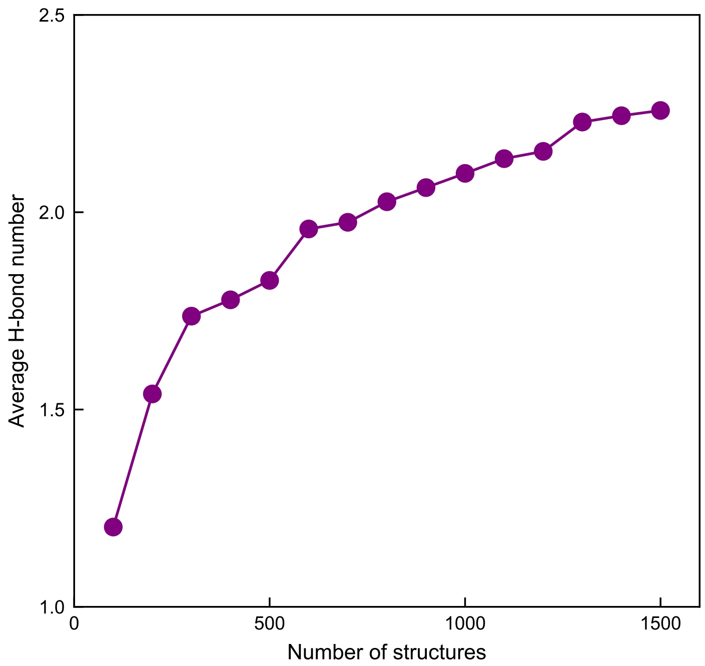

Supplementary Fig. 3: Average hydrogen-bond number in the RSS training set of the water model. This plot illustrates that, as iterations proceed, the model progressively generates more diverse hydrogen-bonded environments, which are subsequently incorporated into the training set. Source data are provided as a Source Data file.

Supplementary References

S1. Pickard, C. J. & Needs, R. J. High-pressure phases of silane. *Phys. Rev. Lett.* **97**, 045504 (2006).

S2. Pickard, C. J. & Needs, R. J. Ab initio random structure searching. *J. Phys.: Condens. Matter* **23**, 053201 (2011).

S3. Packwood, D. *et al.* A universal preconditioner for simulating condensed phase materials. *J. Chem. Phys.* **144**, 164109 (2016).

S4. Bernstein, N., Csányi, G. & Deringer, V. L. De novo exploration and self-guided learning of potential-energy surfaces. *npj Comput. Mater.* **5**, 99 (2019).

S5. Pickard, C. J., Martinez-Canales, M. & Needs, R. J. Decomposition and terapascal phases of water ice. *Phys. Rev. Lett.* **110**, 245701 (2013).

S6. Ong, S. P. *et al.* Python Materials Genomics (pymatgen): A robust, open-source python library for materials analysis. *Comput. Mater. Sci.* **68**, 314–319 (2013).

S7. Togo, A., , S., Kohei & and Tanaka, I. Spglib: A software library for crystal symmetry search. *Sci. Technol. Adv. Mater., Meth.* **4**, 2384822 (2024).

S8. Kühne, T. D., Krack, M., Mohamed, F. R. & Parrinello, M. Efficient and accurate Car-Parrinello-like approach to Born-Oppenheimer molecular dynamics. *Phys. Rev. Lett.* **98**, 066401 (2007).

S9. Kühne, T. D. *et al.* CP2K: An electronic structure and molecular dynamics software package - Quickstep: Efficient and accurate electronic structure calculations. *J. Chem. Phys.* **152**, 194103 (2020).

S10. Goedecker, S., Teter, M. & Hutter, J. Separable dual-space gaussian pseudopotentials. *Phys. Rev. B* **54**, 1703–1710 (1996).

S11. Perdew, J. P., Burke, K. & Ernzerhof, M. Generalized gradient approximation made simple. *Phys. Rev. Lett.* **77**, 3865–3868 (1996).

S12. Thompson, A. P. *et al.* LAMMPS - a flexible simulation tool for particle-based materials modeling at the atomic, meso, and continuum scales. *Comp. Phys. Comm.* **271**, 108171 (2022).

S13. Cheng, B., Engel, E. A., Behler, J., Dellago, C. & Ceriotti, M. Ab initio thermodynamics of liquid and solid water. *Proc. Natl. Acad. Sci. U. S. A.* **116**, 1110–1115 (2019).

S14. Chen, Z., Berrens, M. L., Chan, K.-T., Fan, Z. & Donadio, D. Thermodynamics of water and ice from a fast and scalable first-principles neuroevolution potential. *J. Chem. Eng. Data* **69**, 128–140 (2024).

S15. Stukowski, A. Visualization and analysis of atomistic simulation data with OVITO–the Open Visualization Tool. *Model. Simul. Mater. Sci. Eng.* **18**, 015012 (2009).

S16. Michaud-Agrawal, N., Denning, E. J., Woolf, T. B. & Beckstein, O. MDAnalysis: A toolkit for the analysis of molecular dynamics simulations. *J. Comput. Chem.* **32**, 2319–2327 (2011).

S17. Gowers, R. *et al.* MDAnalysis: A Python package for the rapid analysis of molecular dynamics simulations. in *Proc. of the 15th Python in Science Conference* 98–105 (2016).

S18. Smith, P., Ziolek, R. M., Gazzarrini, E., Owen, D. M. & Lorenz, C. D. On the interaction of hyaluronic acid with synovial fluid lipid membranes. *Phys. Chem. Chem. Phys.* **21**, 9845–9857 (2019).

S19. Gardner, J. L. A. `graph-pes`: train and use graph-based ML models of potential energy surfaces. `https://github.com/jla-gardner/graph-pes` (2024). Version 0.0.25.

S20. Batzner, S. *et al.* E(3)-equivariant graph neural networks for data-efficient and accurate interatomic potentials. *Nat. Commun.* **13**, 2453 (2022).

S21. Park, Y., Kim, J., Hwang, S. & Han, S. Scalable parallel algorithm for graph neural network interatomic potentials in molecular dynamics simulations. *J. Chem. Theory Comput.* **20**, 4857–4868 (2024).

S22. Deringer, V. L., Pickard, C. J. & Csányi, G. Data-driven learning of total and local energies in elemental boron. *Phys. Rev. Lett.* **120**, 156001 (2018).
